# *In vitro* Induction of *Entamoeba histolytica* Cyst-like Structures from Trophozoites

**DOI:** 10.1371/journal.pntd.0000607

**Published:** 2010-02-16

**Authors:** Hugo Aguilar-Díaz, Martha Díaz-Gallardo, Juan P. Laclette, Julio C. Carrero

**Affiliations:** 1 Department of Immunology, Instituto de Investigaciones Biomédicas, Universidad Nacional Autónoma de México, Ciudad de México, México; 2 Department of Developmental Genetics and Molecular Physiology, Instituto de Biotecnología, Universidad Nacional Autónoma de México, Morelos, México; George Washington University, United States of America

## Abstract

Inhibition of encystment can be conceived as a potentially useful mechanism to block the transmission of *Entamoeba histolytica* under natural conditions. Unfortunately, amoeba encystment has not been achieved *in vitro* and drugs inhibiting the formation of cysts are not available. Luminal conditions inducing encystment *in vivo* are also unknown, but cellular stress such as exposure to reactive oxygen species from immune cells or intestinal microbiota could be involved. A role for certain divalent cations as cofactors of enzymes involved in excystment has also been described. In this study, we show that trophozoite cultures, treated with hydrogen peroxide in the presence of trace amounts of several cations, transform into small-sized spherical and refringent structures that exhibit resistance to different detergents. Ultrastructural analysis under scanning and transmission electron microscopy revealed multinucleated structures (some with four nuclei) with smooth, thick membranes and multiple vacuoles. Staining with calcofluor white, as well as an ELISA binding assay using wheat germ agglutinin, demonstrated the presence of polymers of *N*-acetylglucosamine (chitin), which is the primary component of the natural cyst walls. Over-expression of glucosamine 6-phosphate isomerase, likely to be the rate-limiting enzyme in the chitin synthesis pathway, was also confirmed by RT-PCR. These results suggest that *E. histolytica* trophozoites activated encystment pathways when exposed to our treatment.

## Author Summary

One possible approach to the control of amoebisis is based on the simplicity of its life cycle involving only humans with no intermediary hosts and reservoirs. The cyst, responsible for the transmission of this parasite, is an ideal target for developing new drugs to eradicate the disease. However, the stimuli that trigger the encystment of *E. histolytica* and the underlying mechanisms are still mostly unknown. Our study describes the *in vitro* induction of cyst-like structures when trophozoites cultures are exposed to hydrogen peroxide and divalent cations. Evidence is provided to support the activation of chitin biosynthesis resulting in these chitin-walled round structures that might be intermediates in the formation of infective, tetranucleated, mature cysts. We believe this represents a significant advance for the study of the encystment process in amoebae, and may lead to strategies directed to control the propagation of this parasite.

## Introduction

The intestinal parasite *Entamoeba histolytica*, the causative agent of amoebiasis, is estimated to infect 50 million people annually, mainly in developing countries where is a major source of morbidity and mortality [1]. Humans are infected by the amoeba through ingestion of water or food containing cysts, the infective stage of the parasite [2],[3]. Once in a host, the cysts become the active living form of the parasite known as trophozoites. These trophozoites live in the mucosal layer of the colon, and occasionally invade other organs. Therefore, encystation and excystation are the two major differentiation events essential for completion of *E. histolytica* life cycle in the human intestine [2],[4].

Encystation is a complex process, involving intracellular rearrangements, consumption of glycogen reserves, formation of ribosome aggregates, changes in gene expression and transcription, protein synthesis and the deposition of a chitin cyst wall [2]–[4]. This chitin wall is made up of a structural homopolymer of β-(1,4)-linked *N*-acetylglucosamine (GlnNAc) units which confers resistance to harmful agents in the environment, thus facilitating the parasite's survival and dissemination [2]–[5]. This homopolymer is not found in vertebrates but is common in fungi, crustaceans and insects [5]–[7]. Encystment also involves two nuclear divisions to produce a 4n genomic content [2], [8]–[10].

Protozoan parasites such as *Giardia lamblia* and *Entamoeba invadens* are readily induced to encyst under *in vitro* conditions [5],[11]. The reptile parasite *E. invadens* and the free-living *Acanthamoeba castellani* can be efficiently induced to encyst through changes in osmotic conditions or carbon starvation in the culture medium [12]–[14]. Encystation in *G. lamblia* can be induced *in vitro* through multiple types of treatments, including the addition of bovine, porcine or human bile to the culture medium, or the introduction of sodium glycocholate plus myristic, oleic or pentadecanoid acids [15]. During *G. lamblia* encystation, the specific activities of five enzymes responsible for the synthesis of *N*-acetylgalactosamine (the outer filaments of the cyst wall) from fructose-6-phosphate increase between 8 and 4000 fold [16]. The enzyme glucosamine-6-phosphate isomerase (Gln6Pi: 2-amino-2-deoxy-D-glucosamine 6-phosphate ketol isomerase, EC 5.3.1.10) is considered the rate-limiting enzyme during the biosynthesis of *N*-acetylgalactosamine for the giardial cyst wall [16]. This enzyme catalyzes the transformation of fructose-6-phosphate to glucosamine-6-phosphate.

In contrast to other protozoan parasites discussed above, the molecular stimuli triggering encystation of *E. histolytica* have remained elusive, in spite of being under intense scrutiny. Nonetheless, cyst-like structures (CLS) have been obtained *in vitro* by exposing axenic trophozoites to diverse stimuli, including a combination of elevated CO_2_ and restricted glucose in the culture medium [17], a mixture of bovine serum, glucose, sodium tetraborate, vitamins, phosphates, CaCl_2_, MnCl_2_, and CoCl_2_ with liver and pancreas extracts [18], and finally, a combination of chitin synthase cofactors, including Mg^2+^, Mn^2+^ and Co^2+^ [19]. The resulting structures exhibited thick polysaccharide walls and were resistant to the action of detergents. However, these structures were mostly uni-nucleated and lacked the internal structures typical of mature cysts. More recently, detergent-resistant structures with two and three nuclei were obtained by culturing trophozoites in TYI-S-33 media containing *Escherichia coli* and *Enterococcus faecalis*, followed by exposure to high CO_2_ tension and histamine [20]. However, no viability assays were conducted and structures with four nuclei, the hallmark of mature *E. histolytica* cysts, were not observed. Recently, a microarray-based study was performed using both clinical isolates (containing amoebas with typical features of cysts) as well as several amoeba strains cultured *in vitro* in order to determine stage-specific gene expression. Among the analyzed genes, 15% were regulated during development, with clear differences in gene expression patterns between stages. These genes were typically associated with virulence and invasion by amoeba, and were enriched in the trophozoite strains relative to the cyst-like amoebas [21].

Here, we report the *in vitro* induction of multinucleated CLS by *in vitro* treatment of *E. histolytica* trophozoites with H_2_O_2_ in combination with divalent cations in the culture medium. These CLS exhibit morphological characteristics of *E. histolytica* cysts, such as spherical shape and refringence, small size, multi-nucleation and vacuolation, deposition of chitin, and resistance to detergents. Furthermore, a considerable increase in the expression of the enzyme Gln6Pi was observed, suggesting that the chitin biosynthesis pathway is activated in the CLS.

## Materials and Methods

### Parasites

*E. histolytica* trophozoites of HM-1: IMSS strain, were grown at 37°C in sterile TYI-S-33 medium supplemented with 10% adult bovine serum, 100 U/ml of penicillin and 100 µg/ml of streptomycin sulfate [22].

*E. histolytica* positive fecal samples were identified by conventional coproparasitoscopic analysis at a pediatric hospital in the Mexican State of Sinaloa. These fecal samples were donated by N. León-Sicairos. Patient's oral and written consents were obtained. Positivity to *E. histolytica* was confirmed through isolation of cysts from the human feces following the Ritchie method [23]. Afterwards, purification of *E. histolytica*'s DNA was carried out using the QIAmp stool kit (QIAGEN, Hilden, Germany). PCR was performed using primers designed for the differential identification of *E. histolytica* and *E. dispar*, as reported previously [24].

### Induction of cyst-like structures

Trophozoites were chilled on ice for 10 min and harvested by centrifugation at 150×g for 7 min at 4°C. Cells (1×10^5^) were resuspended in 50 ml fresh TYI-S-33 media in culture flasks and incubated at 37°C. After 72 h, trophozoites in log phase (approx. 5×10^6^ cells) were added with 10, 20 and 40 µl of a commercial 30% H_2_O_2_ solution already containing the following elements: cadmium 0.02 ppm, cobalt 0.02 ppm, copper 0.02 ppm, iron 0.1 ppm, nickel 0.02 ppm, lead 0.02 ppm, zinc 0.02 ppm, free sulfuric acid 40 ppm, chlorine 0.5 ppm, phosphate 5 ppm and sulfate 2 ppm (Merck, Darmstadt, Germany). The estimated final concentration of H_2_O_2_ added was between 2 and 8 mM, with traces of elements in the order of 10^−10^ ppm. The culture was then incubated at 37°C, and 5 ml aliquots were collected at 2, 4, 6, 8, and 24 h. After treatment, parasites were washed three times with phosphate buffered saline, pH 7.2 (PBS), counted under microscope and resuspended in PBS containing detergents: 0.1% sarkosyl, or 0.5% Triton X-100 or 0.5% sodium dodecyl sulfate (SDS), and allowed to sit for 20 min at room temperature [18],[25]. After three washes as above, the detergent-resistant trophozoites were resuspended in PBS and counted again in a microscope. Henceforward, detergent-resistant structures will be called CLS (cyst-like structures). The conversion rate index was estimated as the percent of parasites that were resistant to SDS and therefore were converted from trophozoites to CLS. This index was determined for each concentration of H_2_O_2_ and incubation time. Three independent experiments were carried out including triplicates for each concentration of treatment solution and incubation time. In addition, the CLS were stained with Lugol's iodine or Malachite green and observed under microscope. Where is not specified, subsequent experiments were carried out using CLS obtained with 4 mM H_2_O_2_ during 6 h, as these resulted the optimal conditions for trophozoites conversion (see Results).

### Viability assays

An esterase activity assay (suggestive of metabolic cellular activity) was performed on triplicates of the CLS samples that were resistant to detergents. The assay tested the ability of CLS to convert fluorescein diacetate (FDA) to fluorescein, a method previously used to determine viability and animal infectivity of *G. lamblia* cysts [26]. In brief, trophozoite cultures were treated with 2, 4 or 8 mM H_2_O_2_ during 2, 6 and 24 h (see above). Afterwards, cells were pelleted, washed three times with PBS and the CLS were selected by incubation under 0.5% SDS for 20 min. After three washings with PBS, CLS were adjusted to 1×10^6^/ml in PBS. Samples of 100 µl were treated with 1.6 µl of FDA stock solution (2.5 µg/µl in acetone; Invitrogen, USA), mixed and incubated at room temperature for 8 min. Percent of viable cells was determined by counting the number of fluorescent cells under a fluorescence microscope with a BP350–460 filter.

### Transmission electron microscopy

CLS resistant to detergents were pelleted as before, washed three times with PBS and fixed in Karnovsky solution (formaldehyde and paraformaldehyde; 1∶1) by incubation for 72 h at 4°C. Cells were subsequently washed and incubated for 1 h with 0.1 M sodium cacodilate followed by addition of several drops 1% Osmium tetroxide in Zelterqust buffer. The cells were dehydrated by gradual alcohol increments and processed for electron microscopy. Ultrathin sections were collected on copper grids and double stained with 5% uranyl acetate in double-distilled water, and with 0.25% lead citrate in 0.1 N NaOH. Cells were observed and photographed in a JEOL 1010 transmission electron microscope.

### Scanning electron microscopy

CLS resistant to detergents were harvested as described before. Cellular pellets were fixed in 10% formaldehyde, kept in this solution over the course of three days and dehydrated as for transmission electron microscopy. Cells were then resuspended in pure acetone, placed in a critical point dryer and mounted in thin sheets. Finally, cells were impregnated with gold particles for 20 min and observed under a JEOL JSM6360LV scanning electron microscope.

### Calcofluor white staining

CLS resistant to detergents were washed three times with PBS. After centrifugation, pellet samples were placed onto microscope slides and several drops of 0.05% calcofluor white M2R (Fluorescent Brightener SIGMA, USA) in distilled water, were added. The sample was incubated for 10 min and the slide was observed under UV light using a fluorescence microscope as above. Same staining protocol was applied to *E. histolytica* cysts isolated from human feces as described above, which were used as positive control of calcofluor white staining.

### Wheat germ agglutinin (WGA)-binding assay

A method previously employed to test for the presence of yeast chitin was used with minor changes [27]. Microtiter plates of polystyrene were coated with 100 µl of 50 µg/ml WGA in water at 4°C overnight. The plates were washed five times with 300 µl of 0.05% PBS-Tween to remove the excess WGA and blocked with 300 µl of 0.4% bovine serum albumin in 50 mM Tris-HCl pH 7.5 (blocking buffer) for 3 h at room temperature. After three washes with 300 µl of PBS-Tween, 1×10^4^ CLS resistant to detergents were added to each well in 100 µl of PBS. Fresh extract from 1×10^4^ untreated trophozoites in 100 µl of PBS obtained by 3 thawing/liquid nitrogen freezing cycles was used as negative control. Unspecific binding was determined in wells with no extracts or cells added with all reagents. Plates were incubated for 2 h at room temperature with slow shaking, then were washed three times using PBS-Tween. Subsequently, 100 µl of 1∶1000 HRP-conjugated WGA (Sigma L-3892, USA) in blocking buffer was added to each well and incubated for 15 min at room temperature (integrity of the structures was monitored by light microscopy throughout the experiment). After five washes, the reaction was developed through the addition of 100 µl TMB (5,5′- tetramethylbenzidine; Aldrich 860336) for 5 min in the dark. Development was halted by adding 1 N H_2_SO_4_. Color development was evaluated at 430 nm in a spectrophotometer. Three independent experiments were done, each by cuadruplicate.

### RNA extraction and RT-PCR

Total RNA was isolated from *E. histolytica* trophozoites, CLS resistant to detergents and cysts obtained from human feces by using TRIZOL (Invitrogen, USA) following the manufacturer's instructions. Purified RNA was stored at 20°C until used. *E. histolytica* cysts were isolated from feces of patients with intestinal amoebiasis following the standard Ritchie method as described above under “Parasites”. RT-PCR reactions were performed on the total RNA using a Super Script III One-step RT-PCR kit (Invitrogen, Carlsbad, CA, USA) following the manufacturer's instructions. Briefly, 0.4 µg of total RNA was used for the reaction, which was performed using a gradient Mastercycler (Eppendorf) with primers designed to target the *E. histolytica* Gln6Pi sequence (TIGR Accession number XM_648225; Table 1). *E. histolytica* mRNA ADP-ribosylation factor (ARF; Accession number XM_648949) was amplified as an internal control [28],[29] (Table 1). RT-PCR products were run in 1% agarose gels and stained with ethidium bromide. Densitometry analysis was done in images obtained in a Gel Logic Imaging System (Kodak) using the program Kodak Molecular Imaging Sofware (Standard Edition).

**Table 1:** RT-PCR primers used in this work.

Target gene	Primer name	Sequence (5′ to 3′)
***gln6Pi***	Gln6Pi-F	ATGTCATCCACAAACGAAAATATTC
***gln6Pi***	Gln6Pi-R	CAATAGACATGGATTTATCATATC
***EhARF***	ARF-F	GTAGGACTTGATGCTGCC
***EhARF***	ARF-R	TCACCATTAGTTGCAC

## Results

### In vitro obtaining of cyst-like structures (CLS)

**Figure 1:**
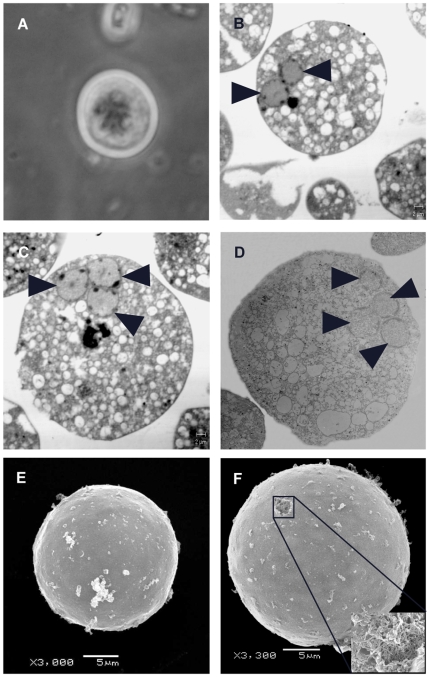
Ultrastructural analysis of *E. histolytica* CLS resistant to detergents. (A) Light microscopy of a CLS stained with Lugol's Iodine showing its spherical shape, multi-nucleation and partial refringence; (B, C and D) Transmission electron microscopy of CLS showing two, three and four nuclei, respectively (arrow heads). High number of cytoplasmic vacuoles are also observed; (E) and (F) Scanning electron microscopy showing the smooth surface, spherical shape and small size of the CLS. Inbox in F shows a magnification of a hole in the surface of a CLS, where overlapping fibers that resembles chitin-fibers in other organisms are observed [6],[7].

Cultures supplemented with a treatment solution of 2 to 8 mM H_2_O_2_ with traces of divalent cations resulted in the efficient conversion of pleomorphic trophozoites into spherical structures with partial refringence that showed resistance to treatment with different detergents, including SDS, sarkosyl and Triton (Figure 1A). The conversion rate, defined as the percent of trophozoites resistant to 0.5% SDS, was dependent on the concentration of the treatment solution and time of incubation. The greatest conversion rates, reaching about 30% of the cells, were produced when the cultures were supplemented with 4 mM H_2_O_2_ and elements present at concentrations of 10^−10^ ppm and incubated for 6 h (Table 2). Two hours post-treatment, 10% of cells had converted, and this proportion gradually increased over the next several hours, decreasing to only 1% after 24 h (Table 2). Trophozoites exposed to treatment solution at H_2_O_2_ concentrations of 2 mM and 8 mM produced notably reduced rates of conversion (Table 2). No conversion was obtained with treatment solution at H_2_O_2_ concentrations less than 2 mM and more to 8 mM (data not shown).

**Table 2:** Detergent-resistance percentage (conversion rate) of *E. histolytica* trophozoites treated with treatment solution at different times.

*Treatment Solution* a	*Incubation Time (h)*	*Viability (FDA) (% ± S.D.)* b	*Conversion rate (% ± S.D.)* b
**10 µl (2 mM)**	2	95±3.6	5±1.5
	6	83±2.5	10±3
	24	20±2.5	0
**20 µl (4 mM)**	2	90±1.7	10±1.5
	6	65±9	30±5.5
	24	10±3.6	2±1
**40 µl (8 mM)**	2	85±12	1±1
	6	20±3.6	5±2
	24	0	0

a Treatment solution (Merck): Hydrogen peroxide 30%, cadmium 0.02 ppm, cobalt 0.02 ppm, copper 0.02 ppm, iron 0.1 ppm, nickel 0.02 ppm, lead 0.02 ppm, zinc 0.02 ppm, free Sulfuric acid 40 ppm, chlorine 0.5 ppm, phosphate 5 ppm, sulfate 2 ppm.

b Three independent experiments were done in triplicates.

Conversion rate: percent of cells that were resistant to 0.5% SDS.

FDA: fluorescein diacetate.

S.D.: Standard deviation.

We performed viability assays on the CLS using FDA, a technique previously used to assay *Giardia* cyst viability [26],[30]. Results were similar for all three tested detergents, and only results of treatment with 0.5% SDS are shown in Table 2. Trophozoites exposed to treatment solution followed by detergent selection showed dose and time dependent mortality. For the concentration of optimal conversion rate (treatment solution at 4 mM H_2_O_2_), approximately 90% of cells exhibited cytoplasmic green fluorescence after 2 h, which decreased to 65% after 6 h of treatment. Mortality continued to increase with length of treatment, reaching 90% cell death after 24 h of exposure followed by detergent selection (Table 2). Trophozoites exposed to treatment solution at 2 mM H_2_O_2_ resulted in lower mortality for all treatment lengths. In contrast, treatment with 8 mM induced high mortality which reached 100% cell death after 24 h. Following studies were then carried out in structures obtained after 6 h of treatment with treatment solution at 4 mM of H_2_O_2_ (maximal conversion rate).

### Morphological characterization

Observation of CLS stained with Lugol's Iodine showed multinucleated CLS with 2 and 3 nuclei (Figure 1A). No structures with 4 nuclei could be observed using this technique. Staining with Malachite green did also not show chromatoid bodies (data not shown). A more detailed morphological characterization was done using transmission electron microscopy. This analysis revealed that approximately 35% of cells possessed two nuclei and 15% possessed 3 nuclei (Figure 1B–C). Cells with four nuclei were much less frequent, and constituted only approximately 5% of the cell population (Figure 1D). In contrast, only approximately 10% of untreated trophozoites were binucleated and we never observed any cells with 3 or 4 nuclei in such cultures.

In addition to the number of nuclei, trophozoites incubated with treatment solution for at least 2 h showed an increase in the number of cytoplasmic vacuoles, particularly in the region adjacent to the plasma membrane. The external surface of the detergent-resistant structures was characterized by scanning electron microscopy. As shown in Figure 1E–F, these structures ranged in size from 10 to 20 µm, showed a smooth surface and spherical shape, and were similar in size and form to other CLS previously obtained *in vitro* [31] as well as *E. histolytica* cysts isolated from clinical specimens. Untreated trophozoites showed typical amoeboid structures approximately 40 to 60 µm in diameter with projecting pseudopods (data not shown). A detergent-resistant structure was found showing an apparent hole in the surface. Further inspection of that zone revealed a network of overlapping fibers (Figure 1F), similar in size and shape to the chitin-fibers found in the exoskeleton of some insects and crustaceans [6],[7]. Together, these results suggest that our treatment is indeed inducing the transformation of trophozoites to CLS.

### Chitin determination

**Figure 2:**
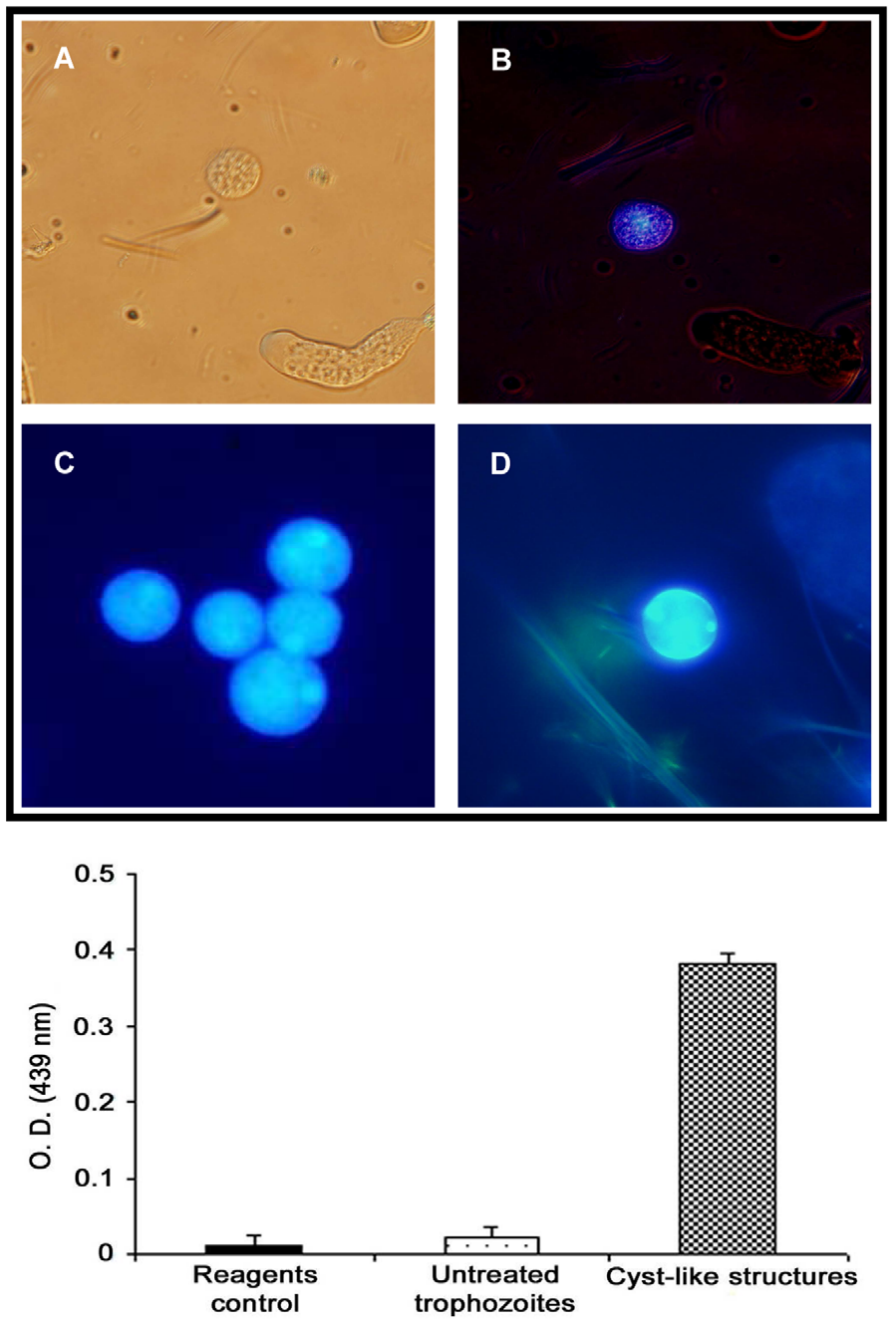
Detection of chitin in *E. histolytica* CLS resistant to detergents by calcofluor white staining (upper panel) and a WGA-binding assay (bottom graphic). (A and B) Light and UV microscopy, respectively, of a mix culture of untreated trophozoites and CLS stained with calcofluor white. CLS is clearly differentiated from trophozoites by the spherical shape and small size. In B, blue fluorescence of CLS under UV contrasts with the absence of fluorescence in the trophozoites 20X; (C and D) Comparison of calcofluor staining of CLS (C) against an *E. histolytica* cyst isolated from human feces (D). In spite of both showing a similar blue-whitish fluorescence, staining of the surface is clearer in the cyst from human feces, whereas staining of some apparently internal structures (arrowheads), probably transporting vacuoles, is evident in the CLS (40X). In the bottom graphic, bars represent the standard deviations.

In order to determine if chitin was present in the CLS, trophozoites treated with 4 mM solution followed by detergent selection were stained with calcofluor white, a fluorescent salt that binds specifically to polysaccharides with β 1–3 and β 1–4 linkages, present in cellulose and chitin. Microscopic examination under UV light showed that the CLS emitted a blue-whitish fluorescence distributed across the surfaces (Figure 2B and 2C); in some instances, fluorescence was also observed inside the CLS probably due to the permeabilization of the structures by the detergent treatment (Figure 2C). Similar patterns of blue-whitish fluorescence with calcofluor were also observed in *E. histolytica* cysts from human feces, used here as positive control (Figure 2D) as well as in previously reported CLS [20]. In contrast, untreated trophozoites mixed with CLS did not show any fluorescence after an identical staining procedure with calcofluor white (Figure 2A and 2B), indicating that chitin was not present. The greater number of fluorescent CLS was observed after incubation of trophozoites treated with 2 mM solution for 6 h (Figure 2B and 2C) corroborating the detergent resistant conversion rates described above.

Another test used to assay the presence of chitin in the surface of the CLS was performed using a WGA-binding assay. Similar to calcofluor white staining, WGA binds specifically to chitin, allowing a semi-quantitative estimation of chitin at the surface when the integral CLS (not extracts) were used in the assay. A significant WGA binding activity was found for the surface of the CLS, in comparison to equivalent samples of untreated trophozoites, for which no WGA-binding was detected (Figure 2; bottom graphic).

### Expression levels of Gln6Pi in CLS

**Figure 3:**
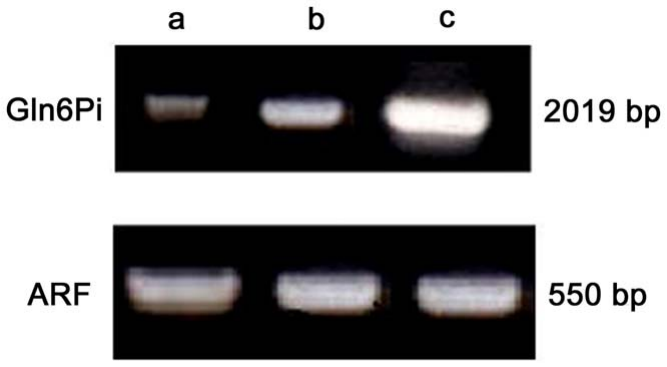
Gln6Pi enzyme mRNA expression levels assessed by RT-PCR in *E. histolytica* CLS resistant to detergents. Total RNA was purified from untreated trophozoites (lane a), cysts isolated from human feces (lane b) and the CLS (lane c) and RT-PCR amplified by using specific oligonucleotides for *E. histolytica* Gln6Pi gene. Clear over-expression of Gln6Pi is observed in CLS (25 folds) respect to the basal expression in untreated trophozoites. Parallel RT-PCR amplification of the ADP-ribosylating factor (ARF) for each sample was used as internal loading control.

The expression levels of Gln6Pi were determined through RT-PCR. This analysis showed a baseline expression of this enzyme in untreated trophozoites. The RNA expression of Gln6Pi was considerably higher than the baseline in cysts isolated from human feces and it was even highest in the obtained CLS. A semi-quantitative analysis by densitometric analysis of the RT-PCR products in gels showed a 25-fold increase in the level of Gln6Pi RNA in the CLS relative to the untreated trophozoites (Figure 3). Gln6Pi RNA levels in cysts from human feces were 7-fold that seen in untreated trophozoites. This result suggests that the isolated CLS may represent an intermediate stage of conversion where the cyst-wall is still developing and requires high levels of enzyme expression, which decrease once the cyst has been completely formed. The RNA levels of *E. histolytica* ARF, used as internal transcriptional and loading control, were similar in all the three samples (Figure 3). These results indicated that Gln6Pi was over-expressed in the CLS as a result of the inductive treatment of the trophozoites. This is consistent with previous results regarding the encystment of *G. lamblia*.

## Discussion

No consistent method for inducing the *in vitro* formation of mature cysts in *E. histolytica* is available. Among the multiple intestinal factors that may be involved in triggering *E. histolytica* encystment, the exposure of trophozoites to reactive oxygen species remained to be tested. Some reports have described high concentrations of oxidizing compounds in the intestinal lumen, generated by the digestive processes, metabolic activity of the microbiota and particularly from infiltrating immune cells such as neutrophils [32]–[34]. Once the trophozoites are released from the cysts and colonize the colonic mucosa to induce mucosal inflammation, these parasites are possibly exposed to all those sources of reactive oxygen species. Moreover, oxygen stress likely increases as trophozoites pass through the digestive system along the ascending and descending colon, where encystment of the trophozoites is thought to take place [2],[3],[29]. In this regard, oxidative stress has been suggested as an environmental stimulus initiating differentiation of *G. lamblia* [15] and H_2_O_2_ has been shown to induce cellular differentiation in other organisms, including fungi and osteoblastic cells [35]–[37]. We found that after addition of small amounts of H_2_O_2_ (perhaps in combination with traces of divalent cations that come together in the commercial preparation) to the culture medium, the trophozoites readily formed spherical, cyst-like, multinucleated structures, resistant to the action of detergents. Maximum conversion rates of approximately 30% were obtained when trophozoites were cultured for 6 hours in the presence of 4 mM of H_2_O_2_ and divalent cations at concentrations of approximately 10^−10^ ppm. Many of those structures were multi-nucleated, with more than 50% showing 2 or 3 nuclei. Notably, up to 5% of cells exhibited 4 nuclei, an unusually high proportion of tetra-nucleated cells for an *in vitro* culture. Infectious *E. histolytica* cysts are known to be tetra-nucleated [9]. To our knowledge, no other treatment produces tetra-nucleated CLS from trophozoites. Recently, Mukherjee and colleagues used DAPI and hematoxylin staining of *E. histolytica* trophozoites cultures and reported that more than 75% of cells were uni-nucleated, 15% were bi-nucleated, and the rest were multi-nucleated; no tetra-nucleated cells were reported [38]. In comparison, an increase in the number of poly-nucleated cells and a decrease in the number of uni-nucleated cells were observed in our CLS, suggesting that nuclear division was induced when the trophozoites were exposed to the treatment solution. As tetra-nucleated CLS were obtained in a relatively short time of treatment (6 hours), it is possible that most or all of these cells were formed by a single nuclear division from bi-nucleated cells. Further research will elaborate this possibility.

Most of the CLS obtained in our study developed a thickened external membrane and were resistant to different detergents, suggesting that they possessed resistant cell walls. Two biochemical assays demonstrated that chitin (a polymer of GlnNAc) was a major constituent of this wall, similar to the walls of mature cysts of *E. histolytica* [39],[40]. Calcofluor white stain with the ability to bind polysaccharides of β-1,4 linkages including chitin, revealed intense fluorescence on the surface and occasionally inside cells, suggesting that chitin polymers are actively transported from the cytoplasm to the cell surface. On the other hand, as WGA binds chitin of *Entamoeba* cysts, including cysts of *E. histolytica*, *E. invadens* and *E. coli* [41], the positive result for this assay with CLS supported the previous result of CLS as chitin-containing structures, strengthening the theory that our treatment triggers the differentiation process of trophozoites into resistant structures. A similar pattern of calcofluor staining was reported in a recent study of *in vitro* induction of *E. histolytica* encystment by co-incubation with enterobacteria and exposure to high CO_2_ tension and histamine [20]. This study reported CLS with features of cysts, including resistance to 0.15% sarcosyl, staining with calcofluor, vacuoles, cytoplasm ribonucleoproteic helices and multi-nucleation up to 3 nuclei. No tetra-nucleated structures were described and the viability of the structures was not assayed. Their descriptions largely match our observations of the CLS, although they described the surfaces of the structured as wrinkled. However, the smooth surface observed in our CLS is consistent with that reported in uni-nucleated CLS obtained by treatment of trophozoites cultures with seven chemical factors [18]. In our case, the viability of CLS was dose and time-dependent as measured by cellular metabolic activity resulting in the conversion of fluorescein diacetate (FDA) to fluorescein (Table 2). In spite of the fact that treatment is aggressive, killing most cells in 24 h, surviving resistant structures can be rescued at early times such as 6 h, where the maximal conversion rate was observed (Table 2). Further studies on the CLS such as *in vitro* excystment and infectivity assays could provide insights about the real viability.

Our results suggest that the reactive forms of oxygen, perhaps in conjunction with the traces of divalent cations coming in the commercial preparation we used, trigger or contribute to triggering a series of cell defense mechanisms, transforming the fragile trophozoites into the resistant CLS. However, the CLS produced by our treatment and those produced in previous studies, may represent intermediary stages between the trophozoite and the mature cysts. This is also supported by the low frequency of tetra-nucleated cells, which represent the final stage of maturity. This possibility cannot be addressed until the development of an adequate animal model that allows testing the infectivity of CLS by oral route.

The molecular mechanisms inducing transformation of trophozoites into CLS under hydrogen peroxide exposure are currently being investigated in our laboratory. In this paper, we addressed the first step in the biosynthesis of the chitin wall of *E. histolytica*. A report on the functional expression of several enzymes involved in the wall biosynthesis of *G. lamblia* cysts, demonstrated a 13 to 182-fold increase of the activity of Gln6Pi for both amination and deamination after treatment of trophozoites with bile [16], suggesting that this enzyme participates in the initial steps of encystment in this parasite [16],[42],[43]. Recent elucidation of the *E. histolytica* genome allowed identification of a possible pathway of chitin biosynthesis [44]. The route is nearly identical to that of *G. lamblia* [45], and includes a cascade of 5 enzymes where Gln6Pi acts in the initial step: the conversion of fructose-6-phosphate into glucosamine-6-phosphate. In turn, glucosamine-6-phosphate is the substrate for a series of reactions culminating with the formation of GlnNAc polymers, which serve as the main component of chitin [44]. In this report, a semi-quantitative estimation of Gln6Pi expression level in resistant CLS (produced after 6 h of treatment with 4 mM H_2_O_2_), demonstrated a 25-fold increase compared to its base-level expression in non-treated trophozoites, suggesting that H_2_O_2_ in combination with traces of divalent cations, is directly or indirectly inducing overexpression of *E. histolytica* Gln6Pi. In contrast, a recent report describing the transcriptional profile of trophozoites under oxidative and nitroxidative stresses [46], did not find over-expression of the gene encoding Gln6Pi. Comparing these data with our results is difficult because they used 1 mM of H_2_O_2_ in cultures where the exact number of trophozoites was not provided, whereas we used higher concentrations (2 to 8 mM) in cultures of 5×10^6^ cells. Furthermore, in the microarray assay, they used parasites incubated with H_2_O_2_ for only 1 h, whereas our studies were conducted with parasites incubated for at least 4 hours. In spite of these differences, the previous study also described the induction of rounded cells like ours, but did not conduct assays to test for resistance to detergents [46].

The presence of divalent cations, in particular Mn^2+^, Mg^2+^ and Co^2+^, is known to promote development of CLS and chitin-like material [19],[31],[47]. The divalent cations used in this study, as well as in other studies, may be required as enzyme cofactors within the metabolic pathways leading to the formation of the cyst wall. These factors may be required for the activity of Gln6Pi and other enzymes involved in amoeba encystment [18], [47]–[49]. Studies in process are aimed to determine the separate contribution of H_2_O_2_ and the divalent cations on the trophozoite's convertion into CLS.

The infectivity of the CLS, and particularly the infectivity of the tetranucleated fraction, remains untested. Unfortunately, with the exception of certain monkey species [50], no animal model is susceptible to infection by oral route, and thus infectivity of CLS cannot be tested for now. In contrast, experimental infection models including Balb/c mice, gerbils, cats and lambs, have been used for infection with cysts and/or trophozites of *G. lamblia*, including cysts obtained *in vitro* [26], [51]–[54]. Our current efforts are focused on achieving oral infection of C3H/HeJ mice, which are susceptible to intracecal infection with virulent trophozoites [28], in order to carry out infection experiments to determine the infectivity and maturity of our CLS.

In conclusion, we have established a reproducible protocol that produces useful amounts of round, multi-nucleated structures of *E. histolytica*, showing chitin envelopes and resistance to detergents. We believe this represents a significant advance for the study of the encystment process in amoeba, and may lead to strategies directed to control the propagation of this parasite.
